# P2X4 Receptor Antagonists Exert Anti-Inflammatory Effects via NLRP3 Inflammasome in a Murine Model of Colitis

**DOI:** 10.1021/acsptsci.6c00336

**Published:** 2026-09-19

**Authors:** Vanessa D’Antongiovanni, Clelia Di Salvo, Matteo Fornai, Laura Benvenuti, Gianfranco Natale, Larisa Ryskalin, Lorenzo Bertani, Elena Lucarini, Lorenzo Di Cesare Mannelli, Carla Ghelardini, Zoltan H. Nemeth, György Haskó, Luca Antonioli

**Affiliations:** † Department of Clinical and Experimental Medicine, 9310 University of Pisa, Pisa 56126, Italy; ‡ Department of Translational Research and New Technologies in Medicine and Surgery, University of Pisa, Pisa 56126, Italy; § Department of Neuroscience, Psychology, Drug Research and Child Health, Neurofarba, Pharmacology and Toxicology Section, 9300 University of Florence, Florence 50139, Italy; ∥ Department of Surgery, Morristown Medical Center, Morristown, New Jersey 07960, United States; ⊥ Department of Anesthesiology, Irving Medical Center, 5798 Columbia University, New York, New York 10032, United States

**Keywords:** DNBS, experimental colitis, inflammatory bowel diseases, intestinal inflammation, NLRP3 inflammasome, P2X4 receptor

## Abstract

Pharmacological inhibition of P2X4 receptors (P2X4Rs) has emerged as a potential therapeutic approach for the management of several immune/inflammatory diseases. However, the contribution of P2X4Rs in the pathophysiological mechanisms underlying colitis-associated intestinal inflammation remain partially unclear. Here, we investigated the anti-inflammatory effects of two novel and selective P2X4R antagonists, NC-2600 and NP-1815-PX, in rats with 2,4-dinitrobenzenesulfonic acid (DNBS)-induced colitis. In addition, the molecular mechanisms of their action were also characterized. Oral treatment with either drug or dexamethasone (DEX) was initiated immediately after DNBS administration and continued for 6 consecutive days. NC-2600 and NP-1815-PX attenuated the body weight loss induced by colitis, whereas DEX had no effect on this parameter. The increase in spleen weight and the macroscopic severity of colonic lesions were reduced by all three treatments. Furthermore, NC-2600, NP-1815-PX, and DEX decreased colonic IL-1β levels and caspase-1 activity. A reduction in tumor necrosis factor levels was observed only following DEX treatment, as neither P2X4R antagonist affected the colitis-associated increase in this cytokine. By contrast, the loss of colonic occludin expression was reversed by NC-2600 and NP-1815-PX, but remained unaffected by DEX. In THP-1 cells, combined exposure to lipopolysaccharide (LPS) and adenosine triphosphate (ATP) increased IL-1β release and the expression of NLRP3, caspase-1, caspase-5, and caspase-8, whereas caspase-4 expression was unchanged. NC-2600 and NP-1815-PX prevented these LPS/ATP-induced alterations. In conclusion, these findings support the potential of selective P2X4R blockade as a therapeutic strategy for intestinal inflammation, possibly through the modulation of NLRP3 inflammasome-related signaling.

## Graphical abstract

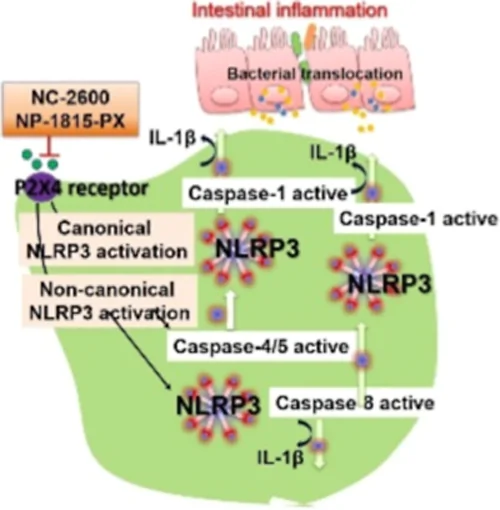

## 1 Introduction

Ulcerative colitis and Crohn’s disease (CD) are the two major forms of inflammatory bowel disease (IBD), a group of chronic disorders affecting the gastrointestinal tract with a relapsing–remitting clinical course. Their incidence and prevalence have progressively increased over the past decades.^1,2^ Due to their progressive and destructive nature, IBDs may lead to several complications, including intestinal stenosis, abscesses, fistulas, extraintestinal manifestations, and an increased risk of colorectal cancer.^3,4^

Although the exact etiology of these conditions remains not fully understood, IBDs are widely recognized to arise from the disruption of the interplay among genetic susceptibility, environmental factors, and intestinal microbiota. Indeed, in genetically predisposed individuals, an aberrant immune response toward commensal microbes leads to early impairment of intestinal epithelial barrier (IEB) integrity, thereby triggering and perpetuating chronic mucosal inflammation.^5^ Moreover, persistent intestinal inflammation may induce long-lasting morphofunctional alterations of the enteric nervous system, along with sensitization of peripheral afferent pathways, contributing to bowel motor dysfunction and visceral pain, reported in approximately 20–30% of IBD patients even after resolution of mucosal inflammation.^6,7^

Unfortunately, approved treatments, including corticosteroids and anti-tumor necrosis factor (TNF) agents, display limited efficacy and safety, with a substantial proportion of patients failing to achieve sustained disease control,^8,9^ underscoring the need to identify novel molecular targets capable of providing effective long-term remission.^10–12^ Accumulating evidence indicates that, under inflammatory conditions, high levels of adenosine triphosphate (ATP) are released by immune and parenchymal cells into the extracellular space. In this context, extracellular ATP acts as a potent pro-inflammatory signaling molecule and participates in autocrine and paracrine circuits regulating cellular metabolism, migration, proliferation, and apoptosis through activation of P2Y and P2X receptors.^13^ Among purinergic receptors, the P2X4 receptor (P2X4R), widely expressed in both innate and adaptive immune cells, has recently emerged as an important modulator of immune and inflammatory responses, as well as enteric sensory pathways, in several pathophysiological conditions, including IBDs.^14^ Accordingly, increased colonic P2X4R expression has been reported and positively correlated with the severity of intestinal inflammation in several experimental models of colitis.^15,16^ Of note, our group has recently demonstrated that two novel P2X4R antagonists, NC-2600 and NP-1815-PX, effectively prevented the development of 2,4-dinitrobenzenesulfonic acid (DNBS)-induced visceral pain, showing superior efficacy compared with dexamethasone (DEX).^17^ Collectively, these findings suggest a relevant contribution of P2X4Rs to IBD pathophysiology and related symptoms, supporting its potential as an innovative therapeutic target. However, to date, a comprehensive evaluation of the effects of P2X4R pharmacological blockade on intestinal inflammation and its underlying molecular mechanisms in experimental colitis is still lacking. Therefore, the aim of the present study was to investigate the role of P2X4R in a rat model of DNBS-induced colitis, as well as the efficacy of its pharmacological blockade by NC-2600 and NP-1815-PX. In addition, complementary *in vitro* experiments were performed to pharmacologically validate the activity of the newly synthesized P2X4R antagonists in a well-established model of purinergic receptor-mediated inflammasome activation, thus providing pharmacological validation prior to their evaluation *in vivo*.

## 2 Materials and Methods

### 2.1 Experiments on Cell Cultures

#### 2.1.1 Cell Culture

Human THP-1 monocytic cells were maintained at 37 °C under humidified conditions with 5% CO_2_. The culture medium consisted of RPMI-1640 supplemented with 10% fetal bovine serum (FBS) and penicillin–streptomycin (100 units/mL).

#### 2.1.2 Cell Treatments

For the experiments, THP-1 cells were seeded in 24-well plates at a density of 5 × 10^5^ cells per well. Monocyte-to-macrophage differentiation was induced by exposure to 0.5 μM phorbol 12-myristate 13-acetate (PMA) for 3 h. Then, the PMA-containing medium was replaced with fresh culture medium and cells were incubated overnight (37 °C, 5% CO_2_).

Differentiated THP-1 cells were initially exposed to lipopolysaccharide (LPS, 1 μg/mL) for 3 h. Without removing the LPS-containing medium, cells were subsequently treated for 1 h with vehicle (0.03% dimethyl sulfoxide, DMSO), NC-2600 (0.03, 0.3, or 3 μM), or NP-1815-PX (0.1, 1, or 10 μM). ATP (5 mM) was then introduced into the same medium, followed by a further 1 h incubation. At the end of the stimulation protocol, cell lysates and culture supernatants were collected for subsequent analyses.

Untreated cells maintained under the same culture conditions served as the controls.

A schematic representation of the stimulation protocol is shown in Figure 1.

**Figure 1:**
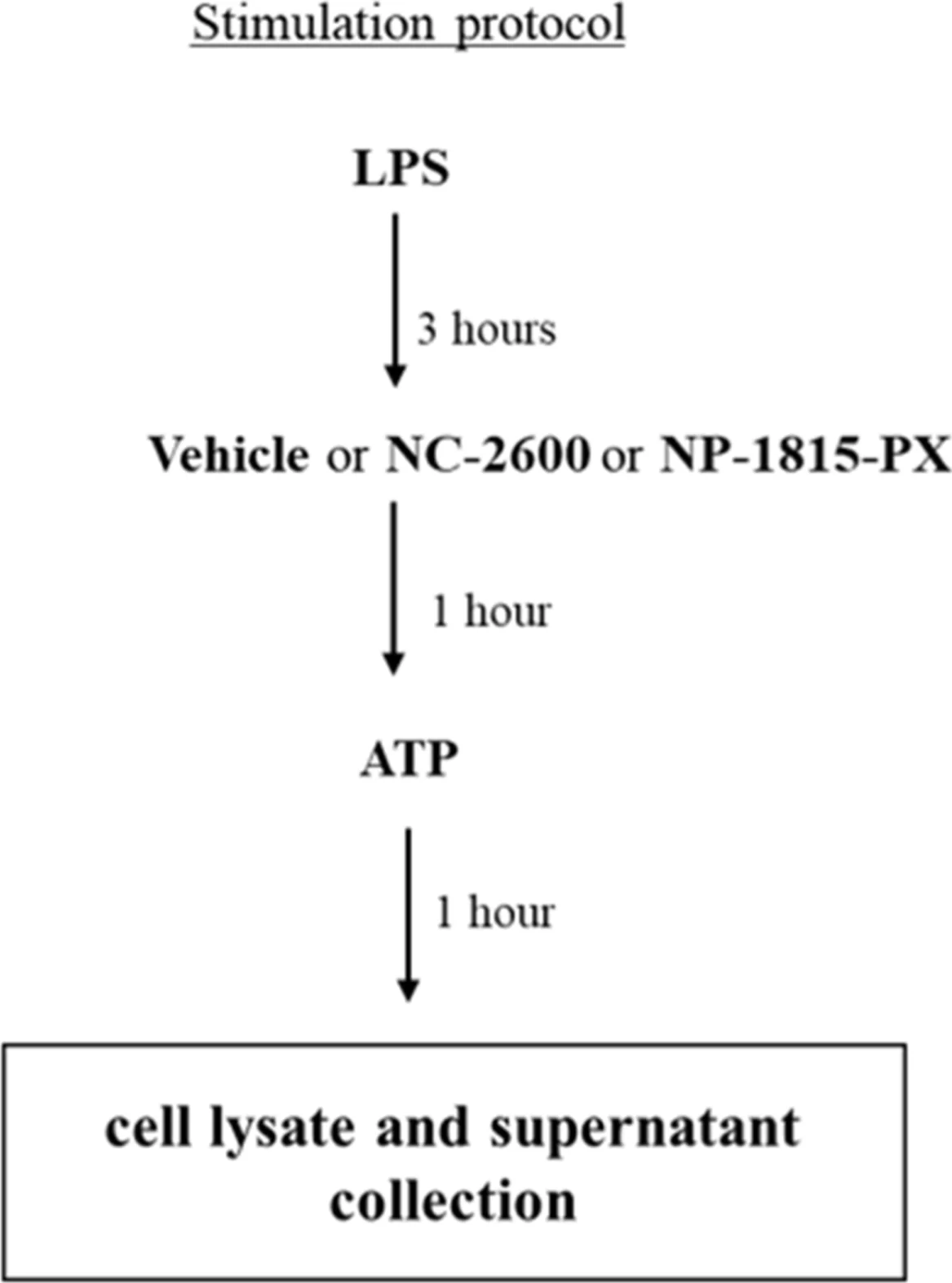
Experimental design of the in vitro study conducted in human THP-1 monocytic cells. Cells were primed with LPS (1 μg/mL) for 3 h and then exposed, without replacing the LPS-containing medium, to vehicle (0.03% DMSO), NC-2600 (0.03, 0.3, or 3 μM), or NP-1815-PX (0.1, 1, or 10 μM) for 1 h. ATP (5 mM) was subsequently introduced for an additional 1 h. At the end of the protocol, cell lysates were used to assess inflammasome components and caspase-1, -4, -5, and -8 expression, while culture supernatants were collected for the measurement of IL-1β release.

The concentration of LPS was selected in accordance with previous studies.^18,19^ ATP (5 mM) was used according to a well-established protocol to induce robust NLRP3 inflammasome activation in THP-1-derived macrophages.^18,20^ Under these experimental conditions, the contribution of P2X4R signaling was assessed by pharmacological blockade with the selective antagonists NC-2600 and NP-1815-PX rather than by selective receptor activation with ATP.

At the end of the stimulation protocol, culture media were collected and centrifuged at 60*g* for 5 min to remove residual cells. The resulting supernatants were used to quantify IL-1β release, whereas cell lysates were analyzed for inflammasome components and the expression of caspases-1, -4, -5, and -8.

#### 2.1.3 Western Blot Analysis

Cell lysates were prepared as previously described^21,22^ and their protein content was determined using the Bradford assay. Equal amounts of protein were separated by electrophoresis on precast 4–20% polyacrylamide gels (Mini-PROTEAN TGX, Bio-Rad) and subsequently transferred onto polyvinylidene difluoride (PVDF) membranes using Trans-Blot Turbo PVDF Transfer Packs (Bio-Rad). Membranes were blocked with 3% bovine serum albumin (BSA) prepared in Tris-buffered saline containing 0.1% Tween 20 (TBS, 20 mM Tris–HCl, pH 7.5, 150 mM NaCl). The membranes were then incubated with primary antibodies against β-actin (ab8227, Abcam), nucleotide-binding oligomerization domain, leucine rich repeat and pyrin domain containing protein 3 (NLRP3) (ab214185, Abcam), apoptosis-associated speck-like protein containing a caspase recruitment domain (ASC) (D2W8U, Cell Signaling), caspase-1 (ab1872, Abcam), caspase-4 (#4450, Cell Signaling), caspase-5 (#46680, Cell Signaling) and caspase-8 (ab227430, Abcam). Anti-mouse (ab97040, Abcam) and anti-rabbit (ab6721, Abcam) secondary antibodies were used as appropriate. Protein bands were detected with enhanced chemiluminescence (ECL) reagents (Clarity Western ECL Blotting Substrate, Bio-rad). Densitometry was performed by IBright Analysis Software.

#### 2.1.4 Measurement of IL-1β Release in THP-1 Cells

IL-1β levels were measured by enzyme-linked immunosorbent assay (ELISA), as previously described.^23^ After cell stimulation, the culture media were collected and centrifuged at 60*g* for 5 min to obtain cell-free supernatants. Aliquots of 100 μL of each supernatant were used for the assay. Results were expressed as a percentage of the values measured in untreated THP-1 control cells.

### 2.2 Experiments on Animals

#### 2.2.1 Animals

Male albino Sprague-Dawley rats, weighing 200–225 g, were employed during this study. Upon arrival at the animal facility, rats were allowed to acclimatize for at least 1 week before the beginning of the experimental procedures. Animals were housed three per cage under controlled environmental conditions (temperature, 22–24 °C; relative humidity, 50–60%; 12 h light/dark cycle) and had unrestricted access to standard laboratory chow and tap water. All procedures involving animals were conducted in compliance with European Directive 2010/63/EU, recognized and adopted by the Italian Government. The experimental protocol was approved by the Ethical Committee for Animal Experimentation of the University of Pisa and by the Italian Ministry of Health (authorization no. 674/2016-PR).

#### 2.2.2 Induction of Colitis and Drug Treatments

Following body weight recording, rats were randomly allocated to the experimental groups with six rats in each. Colitis was induced according to the procedure described previously by Pellegrini et al.^24^ Briefly, rats were placed under short anesthesia with isoflurane (Abbott Laboratories, Pomezia, Italy) and received an intrarectal administration of 2,4-dinitrobenzenesulfonic acid (DNBS, 15 mg in 0.25 mL of 50% ethanol). The solution was delivered through a PE-60 polyethylene catheter introduced 8 cm proximal to the anus. Control rats received an equivalent volume (0.25 mL) of 50% ethanol. Following DNBS administration, rats fully developed established colonic inflammation, which was evident 6 days after DNBS administration.

NC-2600 (3, 10, or 30 mg/kg), NP-1815-PX (3, 10, or 30 mg/kg), or DEX (1 mg/kg) were administered orally once daily for six consecutive days, starting on the day of DNBS injection. DNBS-untreated animals (control group) and DNBS-treated rats (DNBS group) received methocel (drug vehicle) alone. The body weight was recorded daily from the first day of treatment. Animals were euthanized on day 8 (Figure 2).

**Figure 2:**
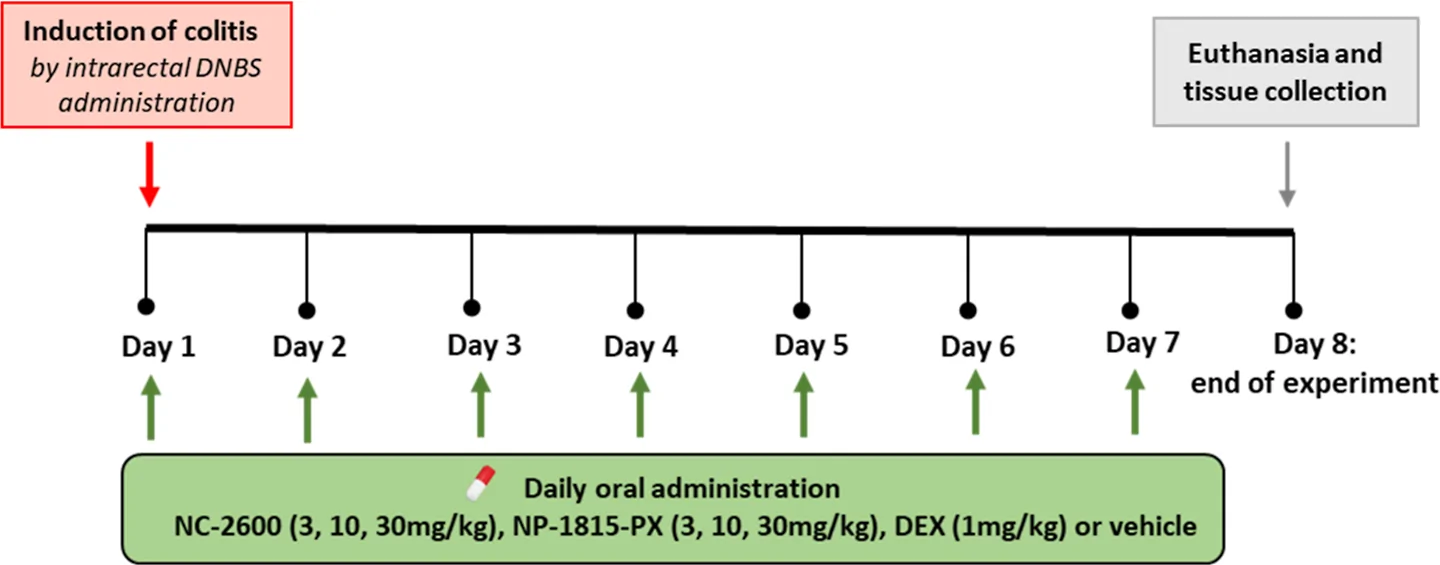
Experimental design of the in vivo study. On day 1, colitis was induced by intrarectal instillation of DNBS. Starting on the same day, rats received vehicle, NC-2600 (3, 10, or 30 mg/kg), NP-1815-PX (3, 10, or 30 mg/kg), or DEX (1 mg/kg) orally once daily for six consecutive days. Animals were euthanized on day 8.

Doses of NC-2600 and NP-1815-PX evaluated in this study (3, 10, and 30 mg/kg) were selected on the basis of preliminary dose-ranging experiments conducted in DNBS-treated rats. In particular, these experiments examined the effects of each compound on food consumption, body weight, spleen weight, and macroscopic colonic damage. On the basis of the outcomes of these preliminary experiments, the 10 mg/kg dose was selected for both NC-2600 and NP-1815-PX to further investigate their effects on inflammasome signaling and tight junction protein expression. The DEX dose was chosen according to a previous study conducted in a rat model of colitis.^25^ Macroscopic and histological scores were assessed on the entire length of the colon, whereas biochemical analyses were conducted on colonic samples obtained from inflamed regions located adjacent and distal to areas of macroscopically evident necrosis.

#### 2.2.3 Assessment of Colitis

Macroscopic colonic damage was evaluated according to a previously described scoring system.^24^ The assessment was based on the evaluation of three distinct parameters: stool consistency, abdominal adhesions, and mucosal ulceration, scored as follows:

•Stool consistency: 0 for formed stools; 1 for loose stools; and 2 for liquid stools.
•Adhesions between the colon and adjacent organs: 0 for absent; 1 for minor; 2 for major.
•Mucosal ulceration: 0 for no lesions; 1 for hyperemia; 2 for ulceration without hyperemia; 3 for ulceration accompanied by inflammation at one site; 4 for ulceration and inflammation at two or more sites; 5 for extensive areas of damage; and 6, extensive damage involving more than 2 cm of the colon.
One additional point was added for each millimeter of the colonic wall thickness. For each animal, all macroscopic parameters were independently assessed by two investigators who were blinded to treatment allocation. The spleen weight was also recorded at euthanasia.

#### 2.2.4 Cytokine Assays

Colonic TNF and IL-1β levels were quantified using ELISA kits (BioSource International, Camarillo, CA), according to previously reported procedures.^23,26^ For this purpose, colonic samples stored at −80 °C were thawed, weighed (40 mg), and homogenized in 0.4 mL of phosphate-buffered saline (PBS; pH 7.2). Then, the homogenates were centrifuged at 13,400*g* for 20 min, and 100 μL aliquots of the resulting supernatants were used for the assays. The concentrations of TNF and IL-1β were expressed as picograms per milligram of tissue protein.

#### 2.2.5 Protein Extraction and Western Blot Analysis

Colons were excised and cleared of fecal material by rinsing with ice-cold PBS, as previously reported.^27,28^ The tissues were finely minced and processed on ice with a Potter–Elvehjem grinder to obtain 20% (w/v) homogenates in TNE lysis buffer containing 50 mM Tris–HCl (pH 7.4), 100 mM NaCl, 0.1 mM EDTA, 1% NP-40, 1% sodium dodecyl sulfate, and 0.1% sodium deoxycholate, supplemented with protease and phosphatase inhibitors. The obtained homogenates were sonicated and subsequently heated at 95 °C for 5 min. Protein content was determined by the Bradford assay. Total lysates were run on 4–20% Criterion TGX Precast Midi Protein Gels (Bio-Rad, Hercules, CA) and then transferred to PVDF membranes (Trans-Blot TurboTM PVDF Transfer packs, Biorad). Membranes were incubated with 3% BSA prepared in TBS-T (TBS, 20 mM Tris–HCl, pH 7.5, 150 mM NaCl) with 0.1% Tween 20, to block nonspecific binding. Primary antibodies against caspase-1 (ab1872, Abcam) and occludin (ab167161, Abcam) were used, followed by the appropriate anti-mouse (ab97040, Abcam) or anti-rabbit (ab6721, Abcam) secondary antibody. Protein bands were detected with ECL reagents (Clarity Western ECL Blotting Substrate, Biorad) and quantified using iBright Analysis Software.

#### 2.2.6 Caspase-1 Activity Assay

Caspase-1 enzymatic activity in colonic tissues was determined using a colorimetric protease assay kit (BioVision Research Products, Mountain View, CA), in accordance with the manufacturer’s instructions. Frozen colon samples were thawed, and 40 mg of tissue was homogenized in 0.4 mL of PBS (pH 7.2). Following centrifugation at 13,400*g* for 20 min, a 100 μL aliquot of each supernatant was used to perform the assay.

### 2.3 Drugs and Reagents

ATP, DEX, DMSO, DNBS, FBS, LPS, PBS, and PMA were obtained from Sigma-Aldrich (St. Louis, MO). NC-2600 and NP-1815-PX were synthesized by Nippon Chemiphar Co., Ltd. For the cell-based experiments, NC-2600 was initially dissolved in sterile DMSO and then diluted in culture medium to reach the required concentration. For the animal experiments, NC-2600 was administered to animals as a solid-dispersion formulation because of its poor aqueous solubility.

### 2.4 Statistical Analysis

Results are reported as mean ± standard error of the mean (SEM). Statistical analyses were conducted using GraphPad Prism 10.0 (GraphPad Software Inc., San Diego, CA). Differences among experimental groups were evaluated by one-way analysis of variance (ANOVA), followed by Dunnett’s multiple-comparisons test. Values of *p* < 0.05 were considered statistically significant.

## 3 Results

### 3.1 *In vitro* Cell Culture Experiments

*In vitro* studies were conducted in THP-1 human monocytic cells to assess whether P2X4R antagonists modulate IL-1β release and the expression of key components associated with the canonical and non-canonical NLRP3 inflammasome pathways, including NLRP3, ASC, caspase-1, caspase-4, caspase-5, and caspase-8.

#### 3.1.1 NLRP3 and ASC Expression in LPS plus ATP-Stimulated THP-1 Cells

THP-1 cells exposed to LPS and ATP exhibited a marked upregulation of NLRP3 (114 kDa), whereas ASC (25 kDa) showed a nonsignificant trend toward increased levels compared with control cells (Figure 3A,B). NC-2600 treatment dose-dependently attenuated the increased expression of both NLRP3 and ASC (Figure 3A,B). In contrast, NP-1815-PX significantly reduced NLRP3 expression in LPS plus ATP-treated cells, while its effect on ASC reached statistical significance only at the highest concentration (Figure 3A,B). Overall, these findings indicate that both P2X4R antagonists attenuate the expression of the key components of the NLRP3 inflammasome pathway.

**Figure 3:**
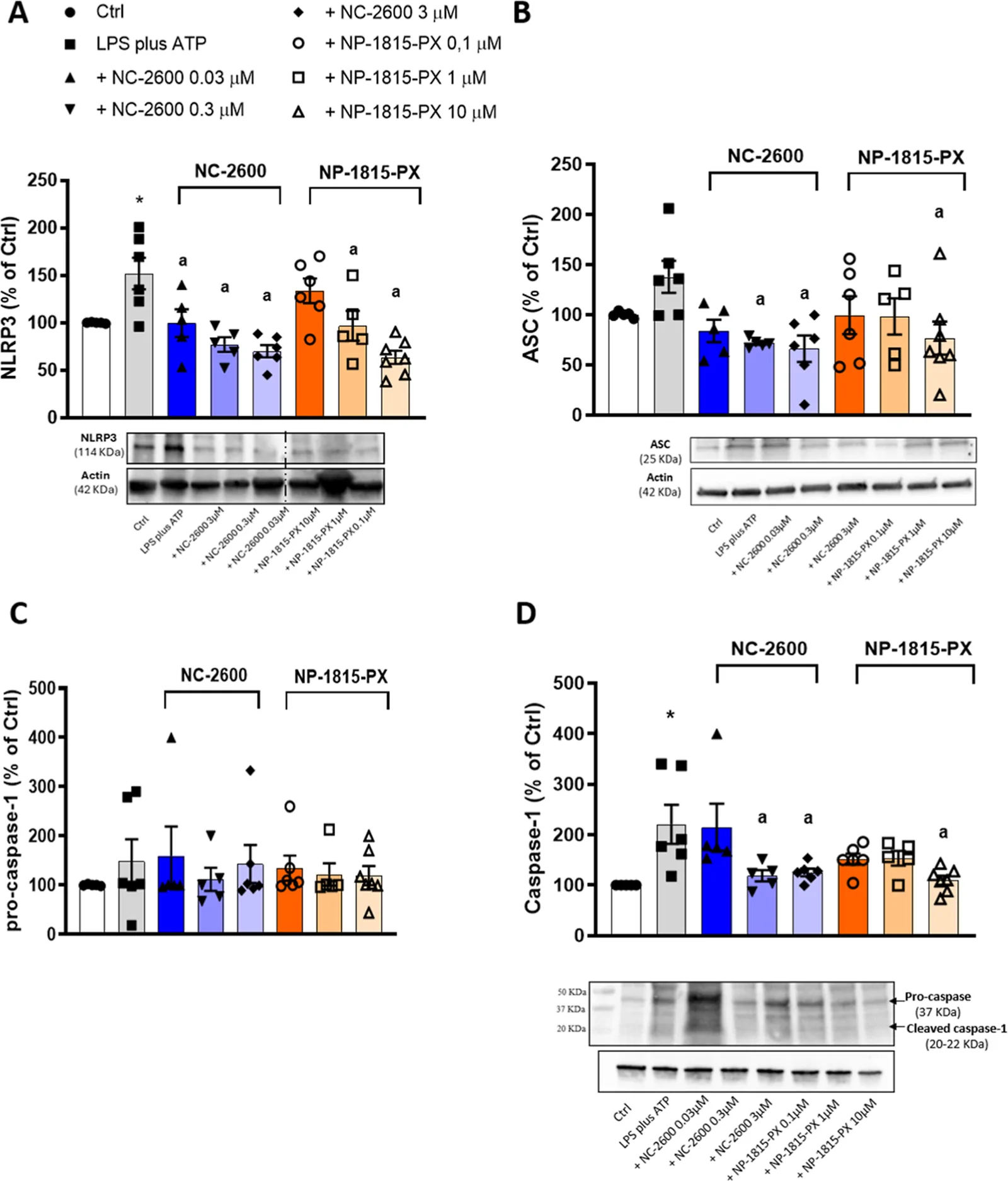
(A–D) Representative Western blots and corresponding densitometric quantification of NLRP3 (A), ASC (B), pro-caspase-1 (C), and cleaved caspase-1 (D) expression in cultured THP-1 cells exposed to LPS and ATP, either in the absence or presence of NC-2600 (0.03, 0.3, or 3 μM) or NP-1815-PX (0.1, 1, or 10 μM). The NLRP3 gel was cropped at the position indicated by the dashed black line. In panel (B), representative immunoblots retain the original sample-loading sequence, with drug concentrations arranged from highest to lowest. The corresponding densitometric data are instead displayed in ascending order of concentration to ensure consistency with the dose presentation used throughout the manuscript. Each column shows the mean ± SEM (*n* = 5–7). Statistical analysis was performed using one-way ANOVA followed by Dunnett’s post hoc test: *p < 0.05 vs THP-1 control cells (Ctrl); ^a^ *p* < 0.05 vs LPS-primed THP-1 cells treated with ATP. Abbreviations: ASC, adaptor protein apoptosis-associated speck-like protein containing caspase-recruitment domain; ATP, adenosine triphosphate; LPS, lipopolysaccharide; NLRP3, nucleotide-binding oligomerization domain leucine rich repeat and pyrin domain containing protein 3.

#### 3.1.2 Pro-Caspase-1 and Cleaved Caspase-1 Expression in LPS plus ATP-Stimulated THP-1 Cells: Canonical NLRP3 Pathway

Neither NC-2600 nor NP-1815-PX altered pro-caspase-1 (45 kDa) expression in THP-1 cells exposed to LPS and ATP (Figure 3C). Conversely, NC-2600 (0.3–3 μM) and NP-1815-PX (10 μM) significantly attenuated the increase in caspase-1 expression in LPS-primed THP-1 cells exposed to ATP (Figure 3D), suggesting that P2X4R antagonists limit the activation of the canonical NLRP3 inflammasome pathway.

#### 3.1.3 Caspase-4, Caspase-5, and Caspase-8 Expression in LPS plus ATP-Stimulated THP-1 Cells: Non-Canonical NLRP3 Pathway

ATP stimulation of LPS-primed THP-1 cells significantly increased caspase-5 and caspase-8 expression, while caspase-4 levels did not significantly differ from control cells (Figure 4A–C). Both NC-2600 and NP-1815-PX significantly counteracted the upregulation of caspase-5 and caspase-8 (Figure 4B,C). These findings indicate that P2X4R blockade also modulates selected components of the non-canonical inflammasome pathway.

**Figure 4:**
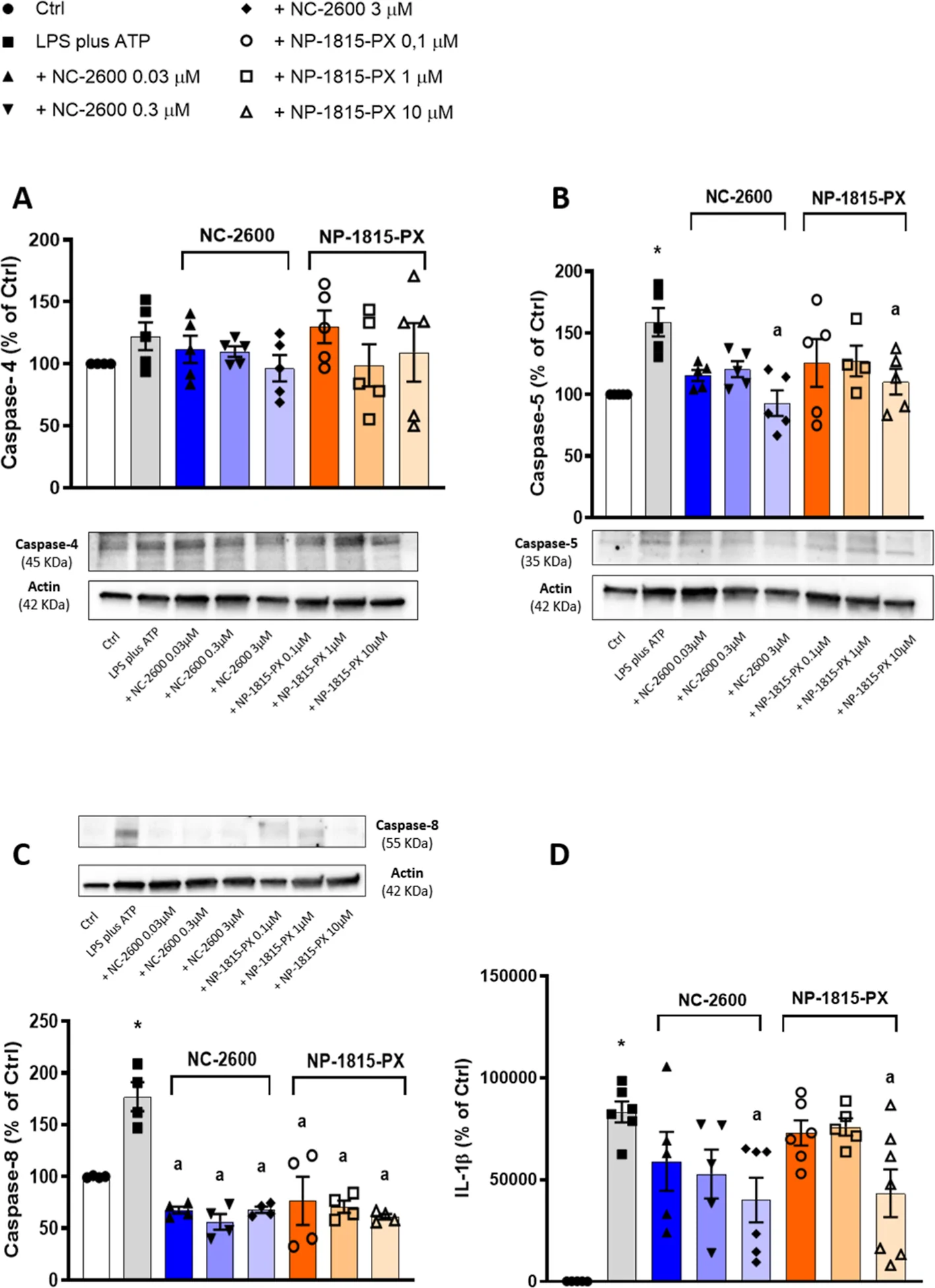
(A–C) Representative Western blots and relative densitometric quantification of caspase-4 (A), caspase-5 (B), and caspase-8 (C) expression in cultured THP-1 cells stimulated with LPS and ATP, either without additional treatment or in the presence of NC-2600 (0.03, 0.3, or 3 μM) or NP-1815-PX (0.1, 1, or 10 μM). Data are presented as the mean ± SEM (*n* = 4–5). (D) IL-1β levels measured in the supernatants of LPS-primed THP-1 cells stimulated with ATP in the presence or absence of NC-2600 (0.03, 0.3, or 3 μM) or NP-1815-PX (0.1, 1, or 10 μM). Data are presented as the mean ± SEM (*n* = 5–7). Statistical significance was determined by one-way ANOVA followed by Dunnett’s post hoc test: *p < 0.05 vs THP-1 control cells (Ctrl); ^a^ *p* < 0.05 vs LPS-primed THP-1 cells treated with ATP. Abbreviations: ATP, adenosine triphosphate; IL-1β, interleukin-1β; LPS, lipopolysaccharide.

#### 3.1.4 IL-1β Release from LPS plus ATP-Stimulated THP-1 Cells

Stimulation of LPS-primed THP-1 cells with ATP significantly enhanced IL-1β release relative to control cells. NC-2600 attenuated this response in a concentration-dependent manner over the range of 0.03–3 μM. By contrast, NP-1815-PX had no significant effect at 0.1 or 1 μM, while a significant reduction was observed at 10 μM (Figure 4D). Overall, these data show the ability of both P2X4R antagonists to attenuate the LPS plus ATP-induced inflammatory response in THP-1 cells.

### 3.2 *In Vivo* Experiments

The *in vivo* experiments were conducted to assess the putative anti-inflammatory effects of P2X4R antagonists in an experimental rat model of colitis and also investigate the underlying molecular mechanisms. In addition, the ability of NC-2600 and NP-1815-PX to restore IEB integrity was assessed. The successful induction of experimental DNBS colitis was evaluated using multiple complementary end points, including body weight loss, spleen weight, colonic macroscopic damage score, and biochemical and molecular markers of intestinal inflammation.

#### 3.2.1 Body and Spleen Weight in Rats with DNBS-Induced Colitis

Six days after DNBS administration, rats exhibited a significant reduction in body weight compared with animals receiving the DNBS vehicle (−36.67 ± 4.41 vs +26.62 ± 3.22%, respectively) (Figure 5A). Treatment with NC-2600 or NP-1815-PX at 3, 10, and 30 mg/kg significantly attenuated the DNBS-induced body weight loss. By contrast, DEX (1 mg/kg) did not produce a significant improvement (Figure 5A).

**Figure 5:**
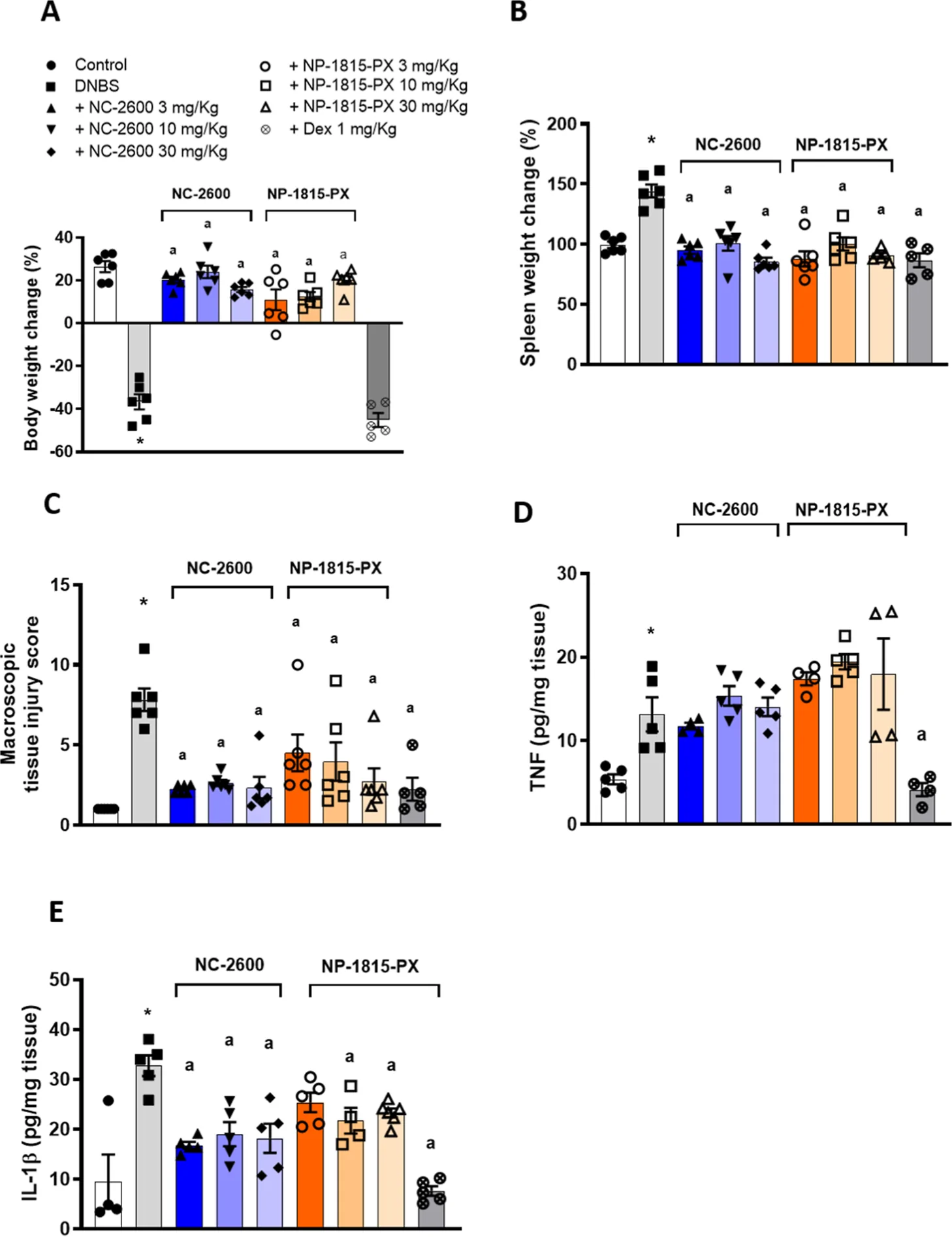
(A–E) Body weight (A), spleen weight (B), macroscopic tissue injury score (C), colonic TNF levels (D), and colonic IL-1β levels (E) in control animals and rats with DNBS-induced colitis receiving vehicle, NC-2600 (3, 10, or 30 mg/kg), NP-1815-PX (3, 10, or 30 mg/kg), or DEX (1 mg/kg). All parameters were evaluated 6 days after DNBS administration. Data are expressed as the mean ± SEM (*n* = 4–6). Statistical comparisons were performed by one-way ANOVA followed by Dunnett’s post hoc test: *p < 0.05 vs the control group; ^a^ *p* < 0.05 vs the DNBS group. Abbreviations: DEX, dexamethasone; DNBS, 2,4-dinitrobenzenesulfonic acid; IL-1β, interleukin 1β; TNF, tumor necrosis factor.

In addition, DNBS animals also displayed a significant increase in spleen weight, with an average increase of 44.33% compared with control animals (Figure 5B). This effect was significantly counteracted by all tested compounds, including NC-2600 (3, 10, and 30 mg/kg), NP-1815-PX (3, 10, and 30 mg/kg), and DEX (1 mg/kg) (Figure 5B).

Administration of these compounds in DNBS-vehicle-treated animals did not significantly affect either parameter (control group; data not shown).

Taken together, these findings indicate that the pharmacological blockade of P2X4R attenuates the severity of DNBS-induced colitis.

#### 3.2.2 Macroscopic Colonic Damage in Rats with DNBS-Induced Colitis

DNBS administration caused colonic wall thickening and ulcerative lesions, accompanied by extensive areas of transmural inflammation. Furthermore, adhesions were frequently observed, and the bowel occasionally appeared dilated, yielding a macroscopic damage score of 7.83 ± 0.61 (Figure 5C). Under these conditions, macroscopic colonic injury was significantly attenuated by all tested doses of NC-2600 and NP-1815-PX, as well as by DEX (Figure 5C). These results demonstrate that both P2X4R antagonists effectively reduce the severity of colonic tissue injury associated with experimental colitis.

#### 3.2.3 Colonic TNF and IL-1β Levels in Rats with DNBS-Induced Colitis

TNF levels were significantly elevated in colonic tissues from DNBS-treated rats compared with controls (12.12 ± 1.78 vs 5.39 ± 0.60 pg/mg tissue, respectively) (Figure 5D). Neither NC-2600 nor NP-1815-PX significantly affected this increase, whereas DEX markedly reduced TNF concentrations (Figure 5D).

DNBS animals also displayed a marked increase in tissue IL-1β levels compared with control rats (32.73 ± 1.88 vs 9.53 ± 5.43 pg/mg tissue, respectively) (Figure 5E). This effect was significantly attenuated by NC-2600, NP-1815-PX, and DEX (Figure 5E).

Overall, these findings suggest that the anti-inflammatory effects of P2X4R antagonists are primarily associated with modulation of the IL-1β response rather than TNF production.

#### 3.2.4 Pro-Caspase-1 and Cleaved Caspase-1 Expression in Rats with DNBS-Induced Colitis

Pro-caspase-1 expression in colonic tissues remained unchanged across the experimental groups (Figure 6A). By contrast, colonic samples from rats with colitis showed significantly higher levels of cleaved caspase-1 expression (p20, the auto-processed fragment of caspase-1) than control animals (Figure 6B). This increase was unaffected by NC-2600 or NP-1815-PX at 10 mg/kg but was significantly attenuated by DEX (Figure 6B).

**Figure 6:**
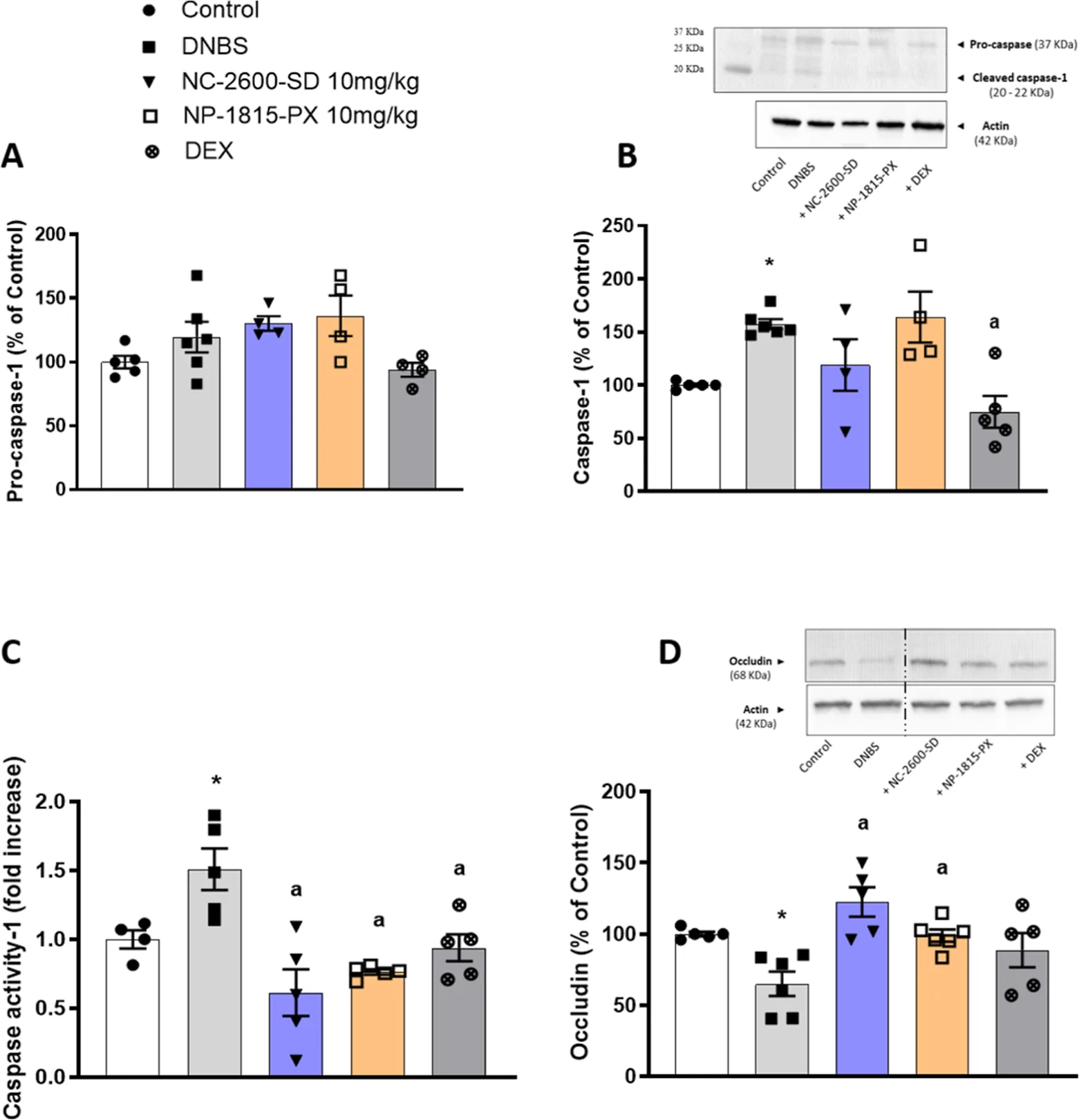
(A–D) Representative Western blots and corresponding densitometric quantification of pro-caspase-1 (A) and cleaved caspase-1 (B) expression in colonic tissues from control animals and rats with DNBS-induced colitis receiving vehicle, NC-2600 (10 mg/kg), NP-1815-PX (10 mg/kg), or DEX (1 mg/kg). Caspase-1 activity measured in the same experimental groups is shown in panel (C). Panel (D) presents representative Western blots and densitometric quantification of occludin expression. The occludin gel was cropped at the position indicated by the dashed black line. Data are expressed as the mean ± SEM (*n* = 4–6). Statistical comparisons were performed by one-way ANOVA followed by Dunnett’s post hoc test: *p < 0.05 versus the control group; ^a^ *p* < 0.05 versus the DNBS group. Abbreviations: DEX, dexamethasone; DNBS, 2,4-dinitrobenzenesulfonic acid.

Taken together, these results suggest that P2X4R antagonists did not affect caspase-1 expression, despite their beneficial effects on intestinal inflammation.

#### 3.2.5 Caspase-1 Activity in Rats with DNBS-Induced Colitis

Caspase-1 activity was significantly higher in colonic tissues from DNBS-treated rats than in control animals (Figure 6C). Treatment with NC-2600, NP-1815-PX, or DEX significantly counteracted the DNBS-induced increase in colonic caspase-1 activity (Figure 6C), suggesting that their anti-inflammatory effects are associated with reduced caspase-1 enzymatic activity.

#### 3.2.6 Occludin Expression in Rats with DNBS-Induced Colitis

Western blot analysis revealed that the expression of occludin, a transmembrane protein localized at tight junctions, was significantly lower in the colonic mucosa of DNBS-treated rats than in control animals (Figure 6D). Rats with colitis treated with NC-2600 or NP-1815-PX showed a significant restoration of occludin expression, whereas no such effect was observed following DEX administration (Figure 6D).

Overall, these findings indicate that the P2X4R blockade counteracts IEB disruption associated with experimental colitis.

## 4 Discussion

In recent years, intensive research has helped clarify the molecular mechanisms underlying IBD pathogenesis, leading to the development of novel biological therapies. However, these agents frequently show limited therapeutic efficacy and may cause adverse effects, including opportunistic infections.^29^ Therefore, the identification of new immunomodulatory and anti-inflammatory therapeutic strategies remains of considerable scientific interest. In this context, increasing attention has been directed toward P2X4R because of its involvement in the regulation of inflammatory responses in several immune-mediated disorders, including IBDs.^12,30–32^ In support to this view, P2X4R has been reported to modulate colitis-associated visceral pain through mechanisms related to inflammatory signaling,^17^ thus suggesting that its pharmacological modulation may represent a promising strategy for the management of IBDs.

On these premises, the aims of the present study were (a) to pharmacologically characterize the activity of two novel and selective P2X4R antagonists, NC-2600, and NP-1815-PX, in a well-established *in vitro* model of purinergic receptor-mediated inflammasome activation and (b) to evaluate their anti-inflammatory efficacy using a rat model of DNBS-induced colitis.

As a first step, we performed a set of *in vitro* experiments on human monocytic THP-1 cells to pharmacologically characterize the biological activity of the newly synthesized P2X4R antagonists in a well-established model of purinergic receptor-mediated inflammasome activation. In particular, we investigated the ability of the tested compounds to counteract the release of IL-1β, considered as a pro-inflammatory mediator that plays a key role in colitis pathogenesis and in the intestinal TH17-related immune response.^33,34^ Of note, the active form of IL-1β is mainly produced by the inflammasome NLRP3, a multimeric protein complex formed by NLRP3, ASC, and pro-caspase-1, whose activation can be triggered by molecular signals originating from tissue damage or invading pathogens.^35^ When the release of IL-1β is mediated by caspase-1, this pathway is designated as “canonical NLRP3 inflammasome activation.” To reproduce key inflammatory stimuli relevant to intestinal inflammation, PMA-differentiated THP-1 cells were exposed to ATP and LPS. ATP was used to examine purinergic receptor signaling in the monocyte/macrophage model,^36–38^ whereas LPS was included as a well-established priming stimulus for the NLRP3 inflammasome and as a mediator associated with impaired intestinal barrier function, features commonly reported in experimental colitis and in patients with IBD.^39,40^ Of note, previous studies have demonstrated that THP-1 monocytes and PMA-differentiated macrophages constitutively express P2X4R, which is functionally active and whose expression is upregulated following LPS stimulation, supporting the use of this cell line to investigate P2X4R-mediated inflammatory responses.^36,41^ Moreover, Huang et al. demonstrated that stimulation of THP-1 cells with LPS followed by ATP further increased P2X4R expression in parallel with the activation of both canonical and non-canonical inflammasome pathways, providing additional evidence that this experimental model is suitable for investigating P2X4R-dependent inflammasome signaling under inflammatory conditions.^20^ Accordingly, treatment of THP-1 cells with LPS and ATP determined the activation of NLRP3 inflammasome pathway, as documented by an increase in NLRP3 and caspase-1 expression along with a massive release of IL-1β. Interestingly, exposure to NC-2600 and NP-1815-PX reduced all the above-mentioned parameters, indicating that their effects may involve modulation of canonical NLRP3/caspase-1/IL-1β inflammasome signaling. Of note, both compounds used in the present study have been pharmacologically characterized as selective P2X4R antagonists and display a high degree of selectivity for P2X4R over P2X7R.^42^ NC-2600 inhibits P2X4R-mediated responses with an IC_50_ of approximately 0.3 μM, whereas its inhibitory potency toward P2X7R is markedly lower (IC_50_ > 30 μM), corresponding to a >100-fold selectivity window.^42^ Similarly, NP-1815-PX exhibits an IC_50_ of 0.26 μM for P2X4R, whereas its IC_50_ for P2X7R is >30 μM, corresponding to a >115-fold selectivity window. Moreover, NP-1815-PX at 1 μM has been shown not to inhibit P2X7R-mediated responses, further supporting its pharmacological selectivity for P2X4R.^42,43^ Therefore, although the high ATP concentration used in the present experimental paradigm does not allow us to completely exclude the contribution of P2X7R or other purinergic pathways to inflammasome activation, the pharmacological profile of the two antagonists supports the interpretation that the observed inhibitory effects are predominantly consistent with P2X4R blockade. Our results are in line with previous studies showing that the pharmacological blockade of P2X4R induces the inhibition of canonical NLRP3 inflammasome activation in neuronal populations of the dorsal horn, rat urothelial cells, and renal tubular epithelial cells exposed to ATP.^20,44–46^ In parallel with the canonical NLRP3 inflammasome activation, a “non-canonical NLRP3 inflammasome activation,” depending on caspase-4 and caspase-5 in humans, whereas caspase-11 in mice, has been reported to play an important role in maintaining the intestinal immune homeostasis as well as in the pathophysiological events underlying bowel inflammation.^47–49^ Moreover, like non-canonical NLRP3 pathway, other studies have identified caspase-8 as a mediator of IL-1β, revealing an additional inflammatory function of caspase-8 beyond its established role in apoptosis.^50,51^ Interestingly, our data extend the effects of P2X4R blockade to components of non-canonical inflammasome signaling. Specifically, NC-2600 and NP-1815-PX decreased caspase-5 and caspase-8 expression following LPS/ATP stimulation of THP-1 cells, whereas neither treatment modified caspase-4 expression. Of note, uncleaved caspase-4 may trigger the release of IL-1β via canonical NLRP3-ASC-caspase-1 signaling.^20,52^ Accordingly, while P2X4R blockade did not modify caspase-4 expression, a possible contribution of uncleaved caspase-4 to the observed reduction in IL-1β release cannot be ruled out. Overall, these *in vitro* observations suggest that P2X4R inhibition affects multiple components of the NLRP3 inflammasome signaling pathway, including both canonical and non-canonical pathways. Of note, these experiments were not designed to provide additional mechanistic insight into P2X4R-mediated inflammasome regulation, which has already been extensively established. Rather, they were intended to pharmacologically validate the activity of the newly synthesized P2X4R antagonists in a relevant inflammatory model, thereby providing a solid rationale for their subsequent evaluation *in vivo*.

Based on our *in vitro* results, the second part of the study was designed to test NC-2600 and NP-1815-PX drugs in a murine model of DNBS-induced colitis. This animal model closely mimics the pathology of CD, including body weight reduction, diarrhea, colonic ulceration and bleeding, goblet cell depletion, and granuloma formation in the intestinal wall,^53,54^ thus providing a useful experimental platform for evaluating the therapeutic potential of novel anti-inflammatory agents. Consistent with these well-established features, DNBS-treated rats in the present study exhibited marked body weight loss, increased spleen weight, severe macroscopic colonic damage, increased colonic inflammatory mediators, enhanced caspase-1 activation, and impaired epithelial barrier integrity, collectively confirming the successful induction of experimental colitis. In addition, the ability of DEX to ameliorate the macroscopic features of colitis and decrease tissue TNF and IL-1β levels further supports the validity of this experimental model, consistent with previous findings.^25,55^ In DNBS-rats, the pharmacological blockade of P2X4R by NC-2600 and NP-1815-PX induced a significant improvement in several colitis-related parameters, including body weight, spleen weight, and macroscopic tissue injury score. In addition, treatment with P2X4R antagonists reduced colonic IL-1β levels in DNBS-treated rats, while tissue TNF concentrations remained unaffected. These findings support a potential role for P2X4R-targeted pharmacological intervention in limiting IL-1β-dependent intestinal inflammation associated with IBD. This observation is consistent with previous evidence indicating that P2X4R contributes to the maintenance of tissue homeostasis during inflammatory conditions, infection, and organ damage.^32^ Indeed, P2X4R is expressed by several innate and adaptive immune cell populations and has been implicated in pathological conditions associated with dysregulated immune activation, including autoimmune and neuroinflammatory disorders.^31^ In addition, our previous study demonstrated a pain-relieving effect of NC-2600 and NP-1815-PX thanks to its ability of modulating NLRP3/IL-1β axis,^17^ thus corroborating the anti-inflammatory effects of tested drugs via NLRP3 inflammasome. To better investigate this aspect, we examined whether NC-2600 and NP-1815-PX could modulate NLRP3 inflammasome activation in DNBS-treated rats, with particular emphasis on caspase-1 expression and enzymatic activity. Although neither antagonist altered caspase-1 protein expression, both compounds significantly reduced its activity in colonic tissue from DNBS-treated animals, thus corroborating *in vitro* evidence on the ability of these novel P2X4R antagonists to inhibit the canonical NLRP3/caspase-1 pathway. Of note, this evidence is in line with previous studies, performed in murine models of diabetic nephropathy, bladder inflammation, and acute kidney injury, showing that P2X4R inhibition was associated with reduced IL-1β release through modulation of NLRP3 inflammasome pathway.^44,45,56^

Of interest, the massive release of IL-1β, besides to contribute to the initiation and amplification of inflammatory responses, promotes the apoptosis of epithelial cells resulting in alterations of the IEB integrity and permeability, features commonly observed in IBDs.^57^ Consistent with these findings, DNBS-treated rats exhibited reduced colonic occludin expression, a tight junction involved in the proper assembly of enterocytes. Interestingly, treatment with both tested drugs prevented the colitis-associated loss of occludin, indicating that P2X4R blockade may contribute to the preservation of IEB integrity. Of note, the molecular basis for the protective effects of these P2X4R antagonists on IEB integrity remains to be fully elucidated. Nevertheless, the preservation of the intestinal barrier may be partly attributable to their ability to modulate the NLRP3/caspase-1/IL-1β axis.

Taken together, the present study expands the current knowledge about the beneficial effects of the P2X4R blockade in modulating immune/inflammatory responses. Our results demonstrated here for the first time that the selective pharmacological targeting of P2X4R attenuates experimental intestinal inflammation and is associated with modulation of both canonical and non-canonical NLRP3 inflammasome signaling pathways (Figure 7). However, genetic P2X4R loss-of-function approaches or direct comparison with P2X7R inhibition will be important to definitively establishing the relative contribution of P2X4R to inflammasome activation under these experimental conditions. In addition, further studies are needed to better characterize the molecular interplay between P2X4R and inflammasomes, thus representing an important focus for future research.

**Figure 7:**
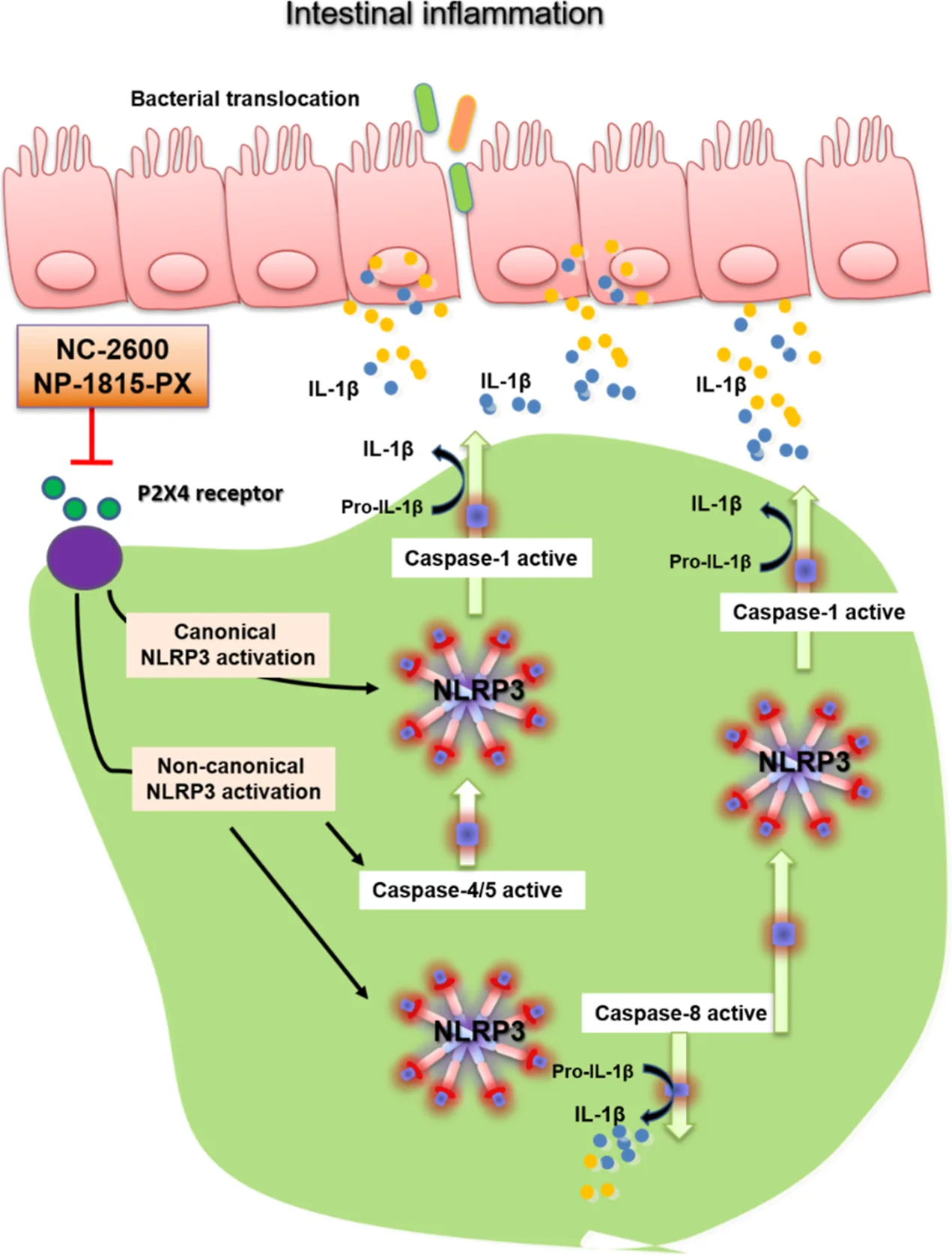
Schematic representation of the proposed effects of NC-2600 and NP-1815-PX on NLRP3 inflammasome-mediated intestinal inflammation. During chronic inflammatory conditions, activation of canonical and non-canonical NLRP3 inflammasome signaling contributes to excessive IL-1β production, which may promote tissue damage and sustain the inflammatory response. Pharmacological targeting of P2X4R with NC-2600 or NP-1815-PX attenuates IL-1β release by interfering with both canonical NLRP3/caspase-1/IL-1β signaling and non-canonical NLRP3, caspase-4, -5, -8, IL-1β signaling, thereby potentially limiting intestinal inflammation. Abbreviations: IL-1β, interleukin-1β; NLRP3, nucleotide-binding oligomerization domain leucine rich repeat and pyrin domain containing protein 3; pro-IL-1β, pro-interleukin-1β.

In conclusion, the present study demonstrates that P2X4R blockade attenuates experimental intestinal inflammation, supporting the potential of selective P2X4R antagonists as candidate therapeutic agents for the treatment of IBD.

## Data Availability

The original contributions presented in the study are included in the article, further inquiries can be directed to the corresponding authors.

## Notes

The experiments have been approved by the Ethical Committee for Animal Experimentation of the University of Pisa and by the Italian Ministry of Health (authorization no. 674/2016-PR).

## Notes

All the authors have read the manuscript and agreed to submit the paper to the journal.
